# Cool Classical EI - A New Standard in EI and Its Many Benefits

**DOI:** 10.1021/jasms.4c00265

**Published:** 2024-07-25

**Authors:** Benny Neumark, Oneg Elkabets, Aviv Amirav

**Affiliations:** † School of Chemistry, Tel Aviv University, Tel Aviv 6997801, Israel; ‡ Aviv Analytical Ltd., 24 Hanagar Street, Hod Hasharon 4527713, Israel

## Abstract

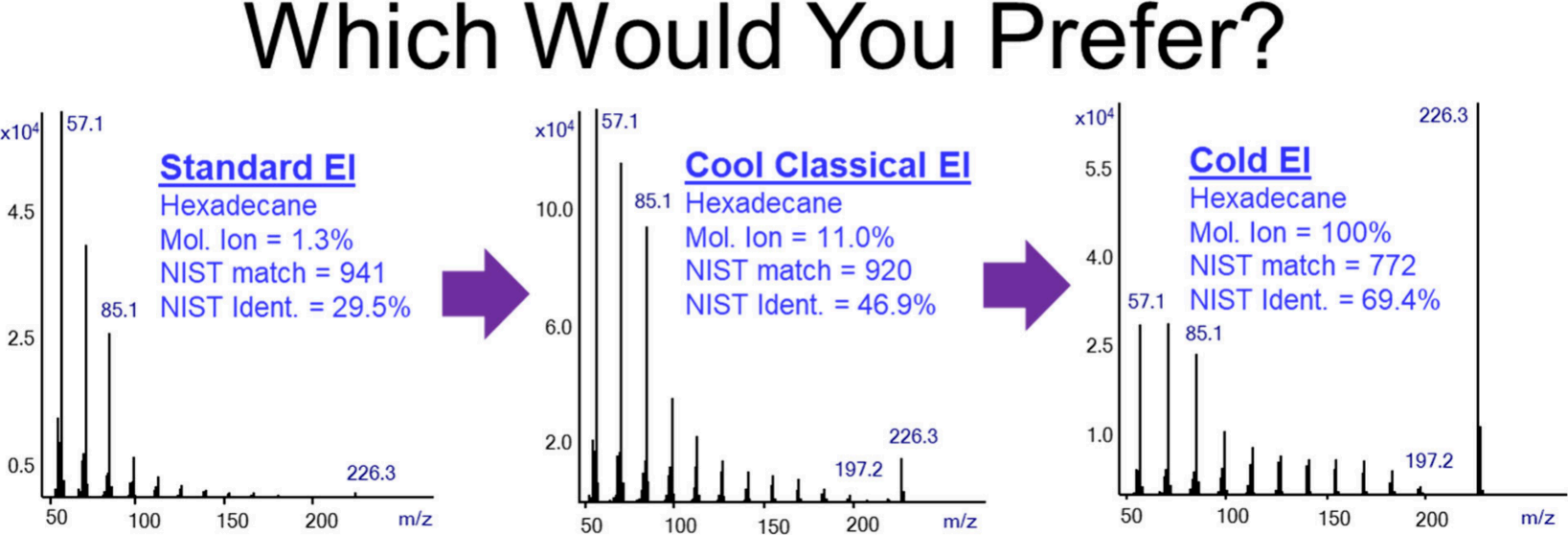

GC-MS with Cold EI improves all of the central GC-MS performance aspects, but it is known mostly for its provision of enhanced molecular ions. This occasionally leads to the misconception that, like chemical ionization, Cold EI is a supplementary ion source to standard EI. However, Cold EI is a highly superior replacement ion source to standard EI. While Cold EI mass spectra are the most informative and fully compatible with mass spectral library (such as NIST) identification, in some cases, the Cold EI mass spectra with their enhanced molecular ions result in a “picture” that is not as one is used to seeing. In this paper, we describe the “Cool Classical EI” mode, which produces classical EI mass spectra like standard EI. The change of Cold EI into the Cool Classical EI mode is software-based, requires no hardware change, and can be achieved even during the analysis. Several mass spectra that were obtained in the Cool Classical EI mode are presented and compared with standard EI and Cold EI mass spectra. In this paper we further demonstrate and discuss several benefits that Cold EI brings that are retained while using Cool Classical EI, including (a) much faster speed of analysis, (b) uniform response, (c) extended range of compounds amenable for analysis, (d) improved sample identification, (e) elimination of ion source related peak tailing, (f) elimination of intraion-source degradation, and (g) better signal-to-noise ratio of the sample compounds.

## Introduction

Gas chromatography–mass spectrometry (GC-MS) is a central technology for unknown compound identification. GC-MS is usually operated with a traditional electron ionization (EI) ion source that produces highly fragmented mass spectra.^1,2^ In addition, EI mass spectra are reproducible, enabling the formation of EI mass spectra libraries such as NIST and Wiley with advanced NIST search and identification algorithm.^3−5^ For an unknown compound to be truly identified, the produced mass spectrum must have a molecular ion, since it is the most characteristic ion, which also provides the ability to confirm the identification via the provision of an elemental formula. Based on our analysis of the NIST MS library, we estimate that approximately 30% of the compounds lack the molecular ion or it is weak (below 2%), and above 400 amu, roughly 50% of the mass spectra lack the molecular ion. Furthermore, the mass spectra found in the NIST EI library are biased toward some enhancement of the molecular ions, since they were produced specifically for the library in working conditions that are not used in everyday practice such as using a relatively low ion source temperature that may result in increased ion source related peak tailing.

The NIST MS search software assigns each result a match factor and an identification probability estimation. The match factor ranges between 0 and 999, and a match factor above 900 is considered excellent, between 800 and 900 it is considered good, and below that it is considered fair or poor. Since the match factor value corresponds to the similarity of the measured mass spectrum to the one found in the library, one would readily accept an identification as correct if the matching is high. The identification probability calculation is more complicated, as it also considers the matching factors of other competing compounds, but it tends to produce much better identification results, particularly when high-mass fragments are more abundant in the measured mass spectrum since they are more characteristic of the measured sample compound. Thus, high identification probability and its being #1 is the best way to ensure proper identification.

GC-MS with Cold EI is based on interfacing the GC and MS with supersonic molecular beams (SMB) and on sample compound ionization during flight through a contact-free fly through ion source for ionization as vibrationally cold sample compounds in the SMB (hence the name Cold EI). Cold EI improves all the central performance aspects of GC-MS, including the provision of enhanced molecular ions that are compatible with isotope abundance analysis for the provision of elemental formula. Cold EI mass spectra also retain the fragment ions for a good library-based identification. Furthermore, Cold EI provides an extended range of low-volatility and thermally labile compounds that are amenable for analysis via its possible use of a high column flow-rate and contact-free ion source. Cold EI was developed by Amirav and his group in 1990,^6,7^ it has been reviewed in refs (8 and 9), a book on GC-MS with Cold EI was recently published,^10^ and its features and applications were published in several papers.^11−25^

GC-MS with Cold EI produces “a lower fit but a better hit” in sample identification, meaning lower matching factors but greater identification probabilities^19^ due to the enhancement of the molecular ion and high mass fragment ions. Based on our discussion with many colleagues, we came to an understanding that even though GC-MS with Cold EI provides much more informative mass spectra and greater confidence in the library identification, many prefer to have the results with higher matching factors and mass spectral similarity to standard EI. This psychological barrier results in the desire to have with Cold EI also a mode that generates standard EI like mass spectra.

In 2008 we published a paper on having classical EI mass spectra in GC-MS with Cold EI that was based on the conversion of the Varian 1200 triple quadrupole instrument into GC-MS with Cold EI.^17^ However, GC-MS with Cold EI has many benefits in addition to its provision of enhanced molecular ions. Thus, in this paper, we shall describe and demonstrate the ability to obtain Classical EI mass spectra while using the Aviv Analytical 5977-SMB GC-MS with Cold EI that it based on the conversion of Agilent 5977B GC-MS into GC-MS with Cold EI. We shall focus on the provision of some limited enhancement of the molecular ions yet without lowering the high similarity to NIST library mass spectra and its matching factors which we name Cool Classical EI (CCEI). Another novel aspect in this article is to show the many benefits of CCEI far beyond its limited enhancement of molecular ions. GC-MS with standard EI systems commonly utilizes a 30 m capillary column and is limited to a column flow rate of 1.2 mL/min. A standard GC-MS analysis usually takes 30–35 min, including cooling in the GC oven for the next injection. Another drawback is the limited range of compounds amenable for GC-MS analysis that directs its users to LC-MS. Thermally labile compounds tend to degrade at the hot GC injector liner, column, and heated transfer line or when interacting with the metallic surface of the hot standard EI ion source. However, GC-MS with Cool Classical EI, like Cold EI with its truly inert fly through ion source,^12^ provides a uniform response to all analytes, including difficult to analyze compounds, and extends the range of compounds amenable for GC-MS analysis.

In this paper, we demonstrate how GC-MS with Cold EI in its CCEI mode outperforms GC-MS with standard EI in a broad range of performance features and provides a new standard for EI in terms of extended range for molecules amenable for GC-MS analysis, speed of analysis, peak tailing elimination, response uniformity, S/N ratio, and much more, and all this while producing detailed mass spectra that are compatible with mass spectra libraries such as NIST and with improved identification.

## Experimental Section

For this research, we used the 5977-SMB GC-MS with Cold EI, which is based on the combination of an Agilent 7890B GC and a 5977 MSD (Agilent Technologies, Santa Clara, CA, USA) combined with the Aviv Analytical SMB interface and its dual-cage fly through ion source (Aviv Analytical, Hod Hasharon, Israel). In GC-MS with Cold EI, the GC column output is mixed with helium makeup gas for a typical total flow of ∼50–60 mL/min combined, while for CCEI it is 10 mL/min. The column output is in front of a supersonic nozzle at the end of a temperature-controlled transfer line. Perfluorotributylamine (PFTBA) can be mixed with the makeup helium flow for system tuning and calibration. The sample compounds inside the helium carrier gas expand from a 100 μm diameter supersonic nozzle into a supersonic molecular beam (SMB) vacuum chamber that is differentially pumped by a Varian Navigator 301 turbo molecular pump (Varian Inc. Torino, Italy) with a 250 L/s pumping speed. The SMB, with its vibrationally cold or slightly cooled sample molecules, passes through a contact-free (thus ultimate-inert) fly through dual cage EI ion source.^12^ The ion source filament generates 70 eV ionizing electrons with 6 mA emission current for the ionization of the analytes seeded in the SMB. The ions are focused using two lenses, deflected 90° by a heated ion mirror, and enter the Agilent 5977 MS for mass analysis. The Agilent triple-axis ion detector detects the ions that exit the quadrupole. MassHunter and ChemStation software were used to process the data.

The Cold EI and CCEI chromatography were performed with a 15 m column with 0.32 mm I.D. and 0.1 μm DB1HT film. The column flow rate was set to 8 mL/min while we added an additional 2 mL/min make up gas flow rate for Cool Classical EI (for the total of 10 mL/min SMB nozzle flow rate) or 46 mL/min additional helium make up gas for the Cold EI experiments. We selected this column, as it provides high flexibility in the GC analysis speed and range of compounds amenable for analysis. The GC oven program started at 50 °C, followed by a gradient of 40 °C/min up to 300 °C with an additional 1.75 min hold time for a total analysis time of 8 min. The injection was done using an Agilent split/splitless injector at 260 °C with a split ratio of 1:9 so that we had 1 ng each hexadecane, methyl stearate, cholesterol, and n-C_32_H_66_ on column. Experiments with Standard EI were performed with an Agilent 7890 GC plus 5977B MSD with its Inert ion source at 300 °C, a 30 m column with DB5MS-UI film, and a 1.2 mL/min column flow rate. The GC oven temperature program was from 50 °C at 10 °C/min up to 320 °C. In Cool Classical EI mode, we used a transfer-line and nozzle temperature of 300 °C. At 10 mL/min combined helium column and makeup gas flow rate, it resulted in sample compounds such as hexadecane with vibrational temperature of 180 °C as we found in the comparison of CCEI data and that of standard EI achieved at 180 °C Inert ion source temperature. For larger compounds the cooling was less and could be only 70–80 °C for n-C_32_H_66_. The main reason for this difference in cooling efficiency is that larger compounds (in terms of number of atoms) have larger internal heat capacity and thus require more cooling collisions with helium at the supersonic nozzle expansion for a certain lower temperature.

## Results

**Figure 1:**
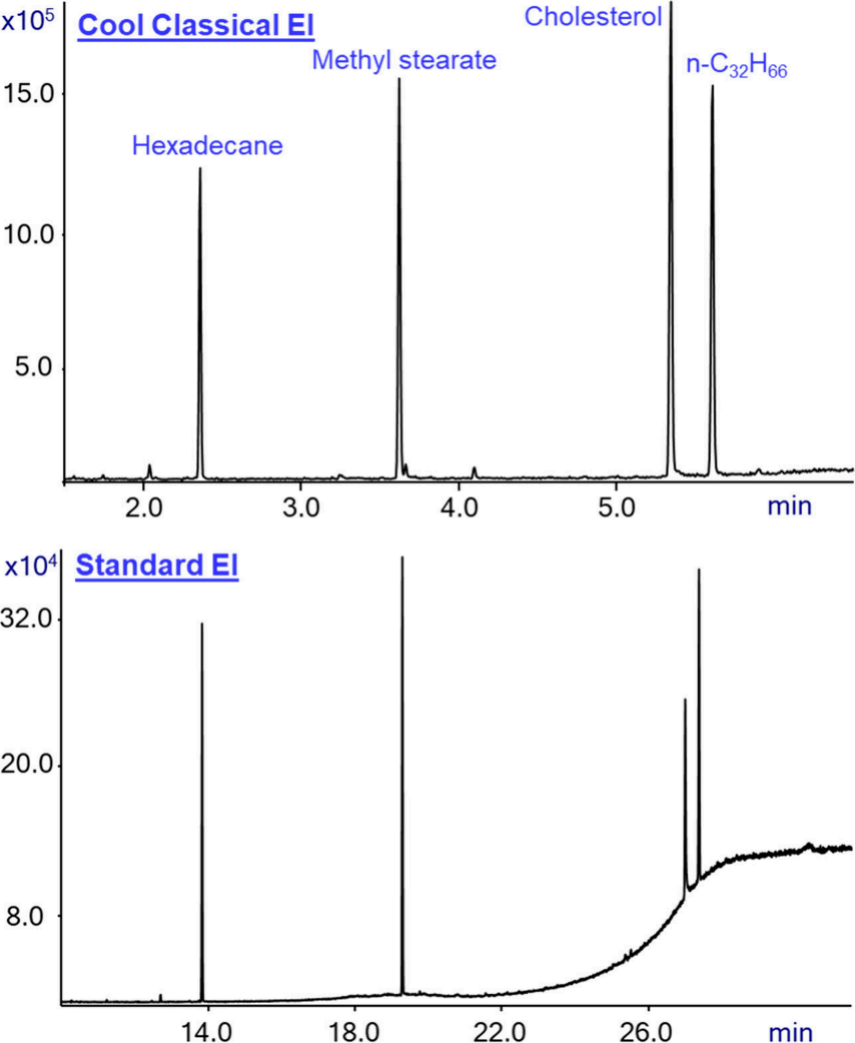
Cool Classical EI (top) and Standard EI (bottom) total ion mass chromatograms are shown from the analysis of our test mix of hexadecane, methyl stearate, cholesterol, and dotriacontane (each at 1 ng on-column) in order of their elution times. Note the faster CCEI analysis time, its uniform response, and lower elution temperatures.

Figure 1 shows the analysis of our test mixture of 1 ng each of hexadecane, methyl stearate, cholesterol, and dotriacontane (n-C_32_H_66_) in both GC-MS with CCEI (upper trace) and GC-MS with standard EI (bottom trace). As shown in the figure, the GC-MS analysis with the Cold EI interface and ion source in its CCEI mode is much faster and takes only 6 min yet retains very good chromatographic separation. Thus, Figure 1 demonstrates the CCEI benefit (a) of having a much faster speed of analysis. It emerges from the use of an 8 mL/min column flow rate that enables a corresponding increase in the GC oven temperature programming rate. The analysis using GC-MS with standard EI is 4 times longer and takes 36 min.

Figure 1 also demonstrates the Cool Classical EI benefit (b) of response uniformity due to its use of a fly through ion source without any reactions with the ion source metallic surface. As shown, the relative cholesterol response is reduced in standard EI but not in CCEI, and also the n-C_32_H_66_ response is not affected in CCEI. The effect of nonuniform response in standard EI is more severe at lower on-column amounts as discussed and further demonstrated in ref (25).

Figure 1 also shows that cholesterol and dotriacontane elute in the standard EI on the column bleed plateau. In contrast, in the CCEI mass chromatogram cholesterol and dotriacontane elute before the onset of column bleed at about 40 °C lower elution temperature than in standard EI. These lower elution temperatures for cholesterol and dotriacontane are the result of using a higher column flow rate of 8 mL/min that reduces the elution temperature by about 20 °C per each factor of 2 higher column flow rate and/or shorter column at a given temperature programming rate.^14^ Accordingly, these lower elution temperatures in CCEI demonstrate its ability to provide its benefit (c) of the extended range of compounds amenable for analysis.

**Figure 2:**
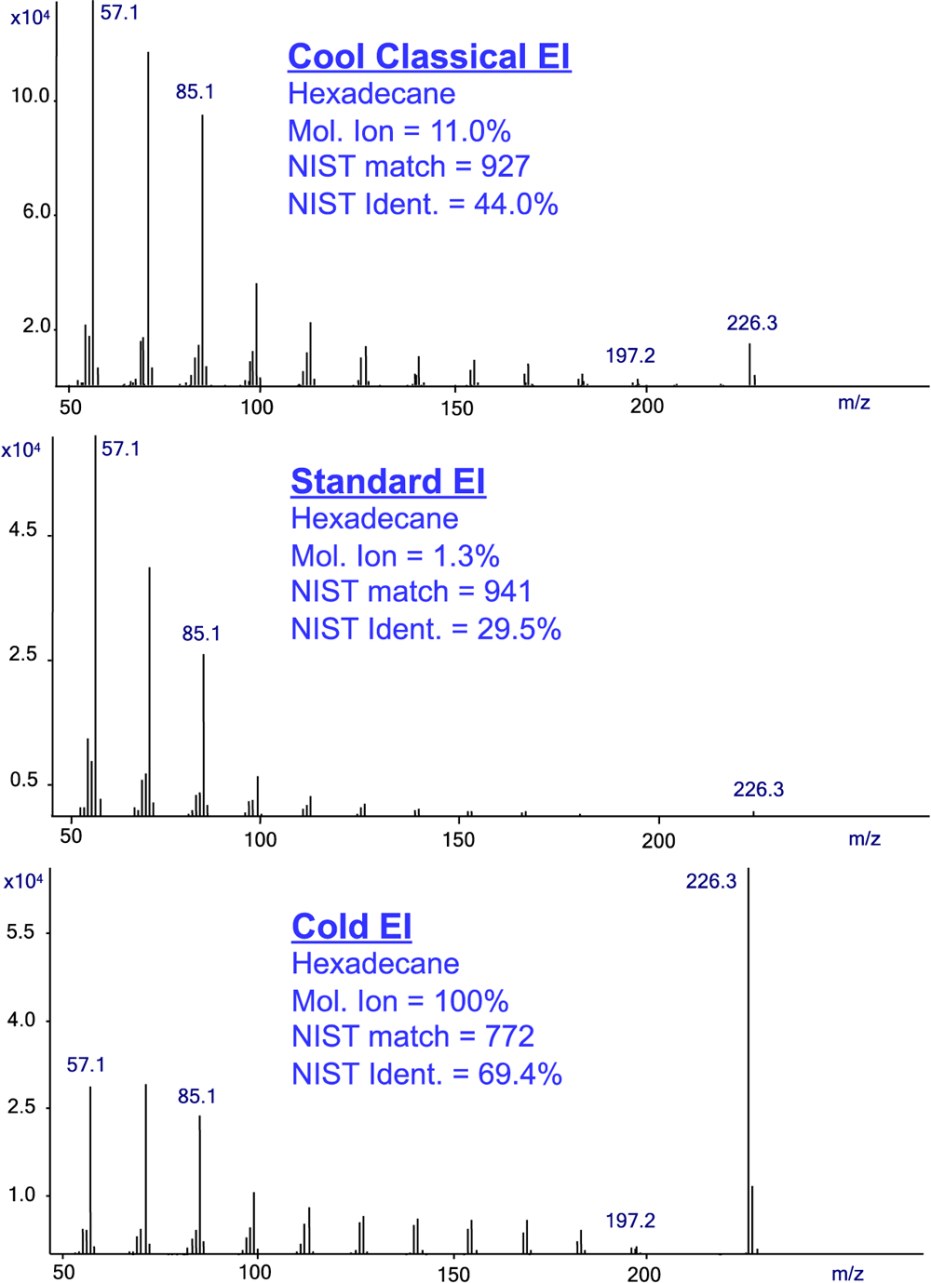
Mass spectra of hexadecane in the Cool Classical EI mode (upper trace), Standard EI (middle trace), and Cold EI (bottom trace). The figure shows the increase of the molecular ion abundance from 1.3% in standard EI to 11.0% in CCEI, and the corresponding increase of the NIST identification probability from 29.5% to 44.0%. In the Cold EI mode, the NIST identification probability is further increased to 69.4%.

In Figure 2 we show the mass spectra that were obtained from the analysis of hexadecane (n-C_16_H_34_). The sample was analyzed using GC-MS with Cold EI in its CCEI mode (upper trace), GC-MS with standard EI (middle trace), and GC-MS with Cold EI in Cold EI mode (bottom trace).

Figure 2 shows that the molecular ion abundance in standard EI is 1.3%, the NIST matching factor is 941, and the identification probability is 29.5%. Using GC-MS with Cold EI in CCEI mode increased the molecular ion abundance from 1.3% to 11.0% and the NIST identification probability from 29.5% to 44.0%, while the matching factor was slightly reduced to 927. Thus, Figure 2 demonstrates that the analysis in CCEI mode retains the mass spectrum that one is used to seeing but delivers far greater identification probability, resulting in a higher confidence in the results. Furthermore, the clearer molecular ions themselves in CCEI confirm the identification. Thus, CCEI provides another benefit (d) of improved identification in comparison with standard EI.

Figure 2 also demonstrates that the molecular ion abundance is increased to 100% when using the Cold EI mode and the NIST identification probability is increased in Cold EI even further to 69.4%. We also note that in both CCEI and Cold EI modes, the mass spectra are fully compatible with the NIST MS search software^3−5^ since, in both cases, the mass spectra retain all the lower mass fragment ions. Despite the match factors in CCEI and Cold EI being lower than those in the standard EI mass spectrum, the identification probability is far better in both cases. We explored standard EI mass spectra at a few Inert ion source temperatures and found that at 180 °C we obtained 11% molecular ion abundance, the same as in CCEI. Accordingly, at 10 mL/min combined column and makeup nozzle flow rate, the supersonic expansion induced vibrational cooling is 120 °C (from 300 °C supersonic nozzle temperature to 180 °C). We note that at 5 mL/min of combined column and makeup nozzle flow rate the supersonic expansion induced vibrational cooling is only about 50–250 °C, which provides mass spectra that are like the standard EI NIST library mass spectra. However, we consider the CCEI mass spectrum such as shown in Figure 2 to approximate the NIST library and standard EI mass spectra well, and yet be more informative and better for identification.

**Figure 3:**
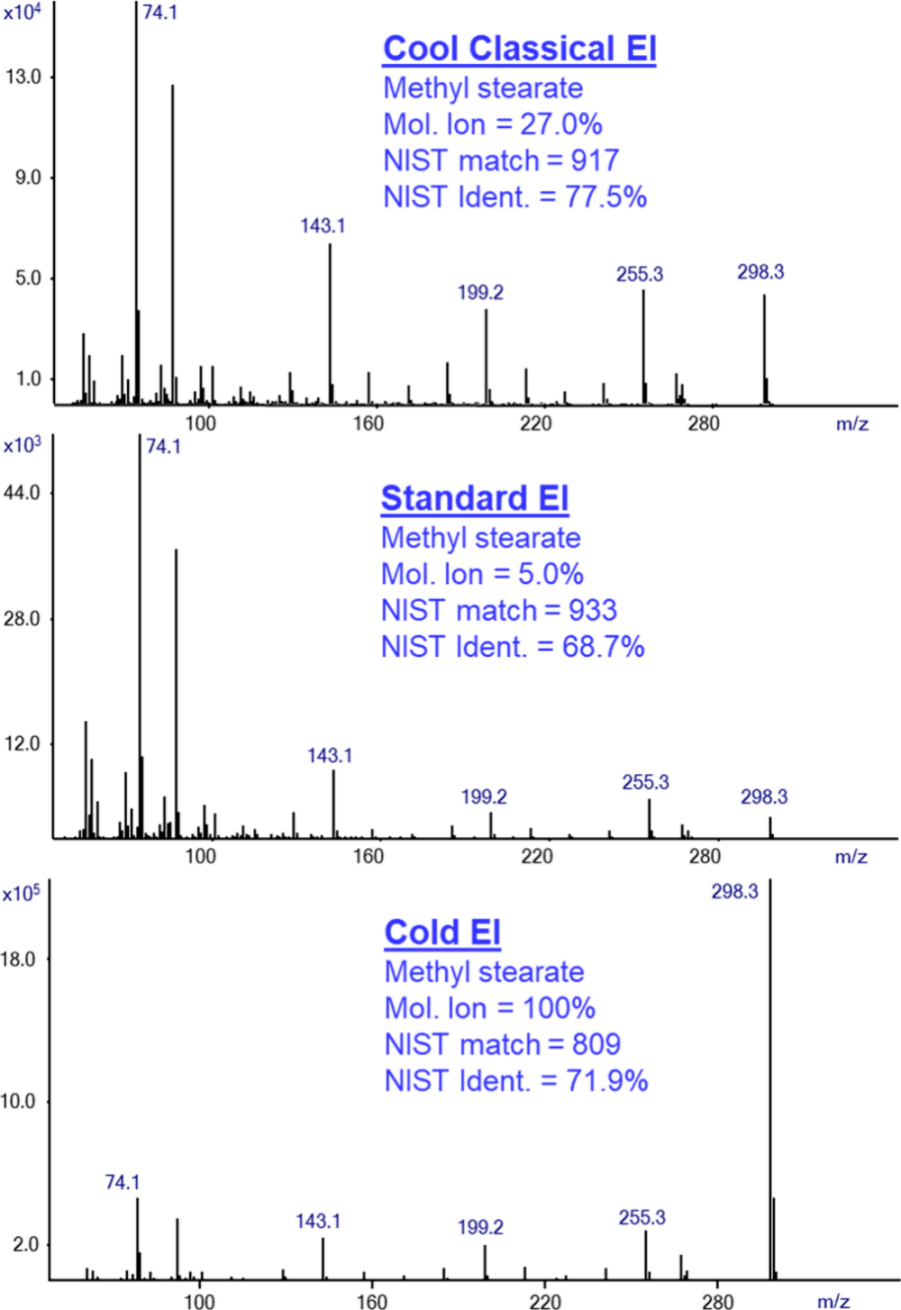
Mass spectra of methyl stearate in the CCEI mode (upper trace), Standard EI (middle trace), and Cold EI (bottom trace). The figure shows the increase of the molecular ion abundance from approximately 5% in standard EI to 27% in CCEI, and the corresponding increase of the NIST identification probability from 68.7% to 77.5%. The NIST identification probability in Cold EI mode is lower than in CCEI mode due to the enhancement of the molecular ion abundance to 100%. However, it is still higher than in standard EI and provides much better confidence in the correctness of identification.

Figure 3 shows the same comparison of EI modes as that obtained from the analysis of methyl stearate. Once again, the analysis using GC-MS with Cold EI in CCEI mode and Cold EI mode provides an enhanced molecular ion, which leads to better results due to improved identification probabilities. While the analysis in Cold EI mode provides a lower identification probability in the NIST mass spectral library than the CCEI mode, it is still higher than the identification probability that was obtained from the standard EI mass spectrum.

However, the enhanced molecular ion with 100% abundance in the Cold EI mode provides much better selectivity and confidence in the identification. As shown in Figure 3, the CCEI mass spectrum combines high similarity to the standard EI mass spectrum yet with some improvement of the molecular ion and high mass fragment ions for better confidence in the identification. The NIST library includes seven main-library and replica mass spectra of methyl stearate with molecular ion relative abundance ranging from 5.3% akin to the standard EI MS shown in Figure 3 up to 35.2%, which is even higher than the MS of CCEI in Figure 3. Thus, while CCEI provides mass spectra like those in the NIST library with a more intense molecular ion, standard EI at 300 °C ion source temperature provides mass spectra with even lower molecular ion abundances than the NIST library mass spectra.

**Figure 4:**
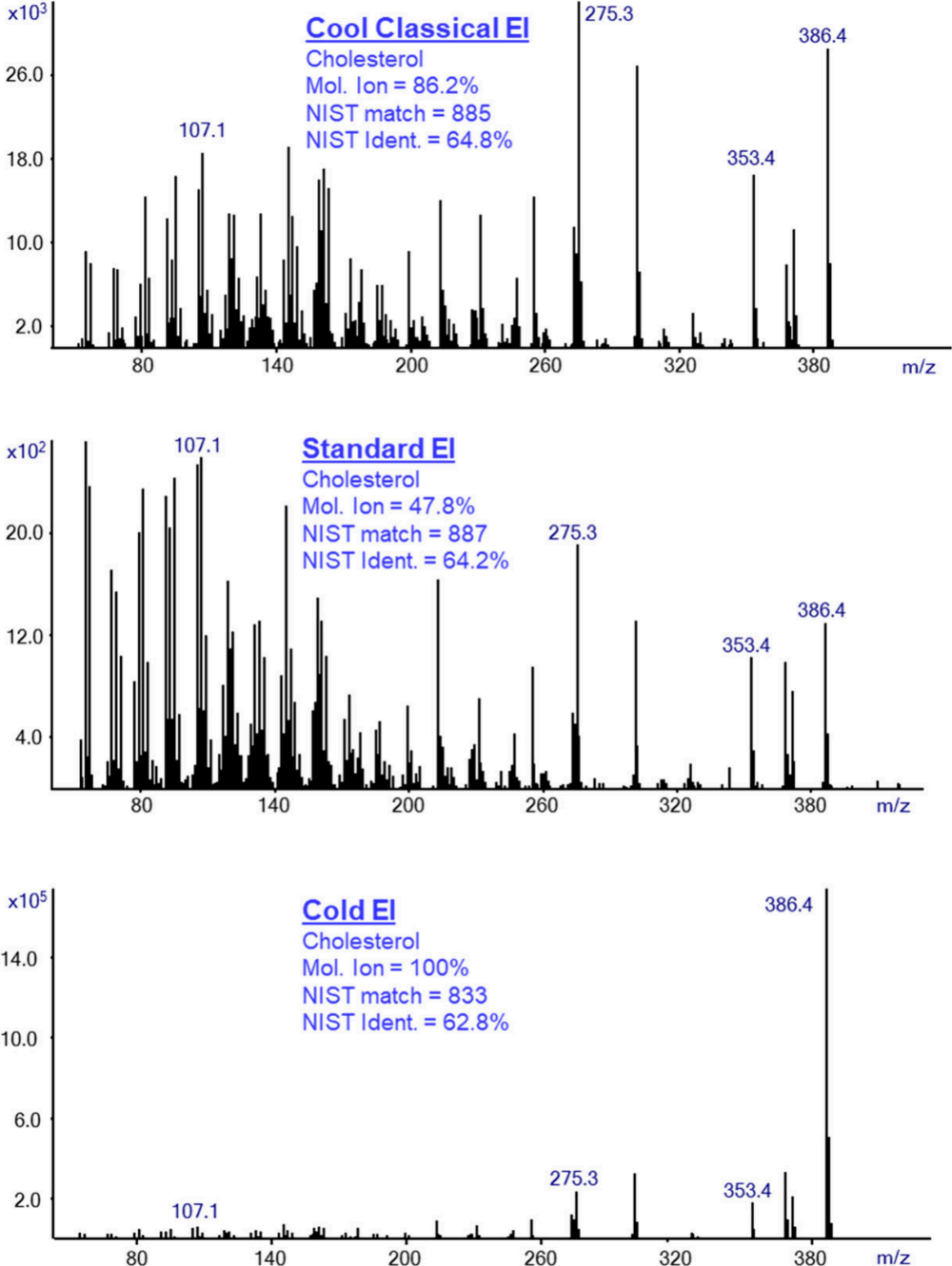
Mass spectra of cholesterol in the CCEI mode (upper trace), Standard EI (middle trace), and Cold EI (bottom trace). The figure shows the increase of the molecular ion abundance from 47.8% in standard EI to 86.2% in CCEI and the increase of NIST identification probability from 64.2% to 64.8%. Note the visual resemblance between the standard EI and CCEI mass spectra. However, with its enhanced molecular ion, the CCEI mode provides greater confidence in the NIST identification. In Cold EI mode, the slightly lower identification probability is due to the enhancement of the molecular ion abundance to 100%.

Figure 4 shows a comparison of the three EI modes for cholesterol. As for methyl stearate, Figure 4 shows some increase of the molecular ion abundance from 47.8% in standard EI to 86.2% in CCEI and a small increase of NIST identification probability from 64.2% to 64.8% in CCEI. We note that the standard EI mass spectrum of cholesterol was obtained after having background subtraction that was required due to extensive column bleed background, unlike in the Cold EI or CCEI mass spectra. As shown, we observed a visual resemblance between the standard EI and CCEI mass spectra, which resulted in almost similar matching factors. However, with its enhanced molecular ion, the CCEI mode provides better confidence in NIST identification. In Cold EI mode, we found a slightly lower identification probability than in CCEI due to the enhancement of the molecular ion abundance to 100%.

However, for cholesterol, all three EI modes provide similar matching factors and identification probabilities, yet they differ in the molecular ion abundance.

**Figure 5:**
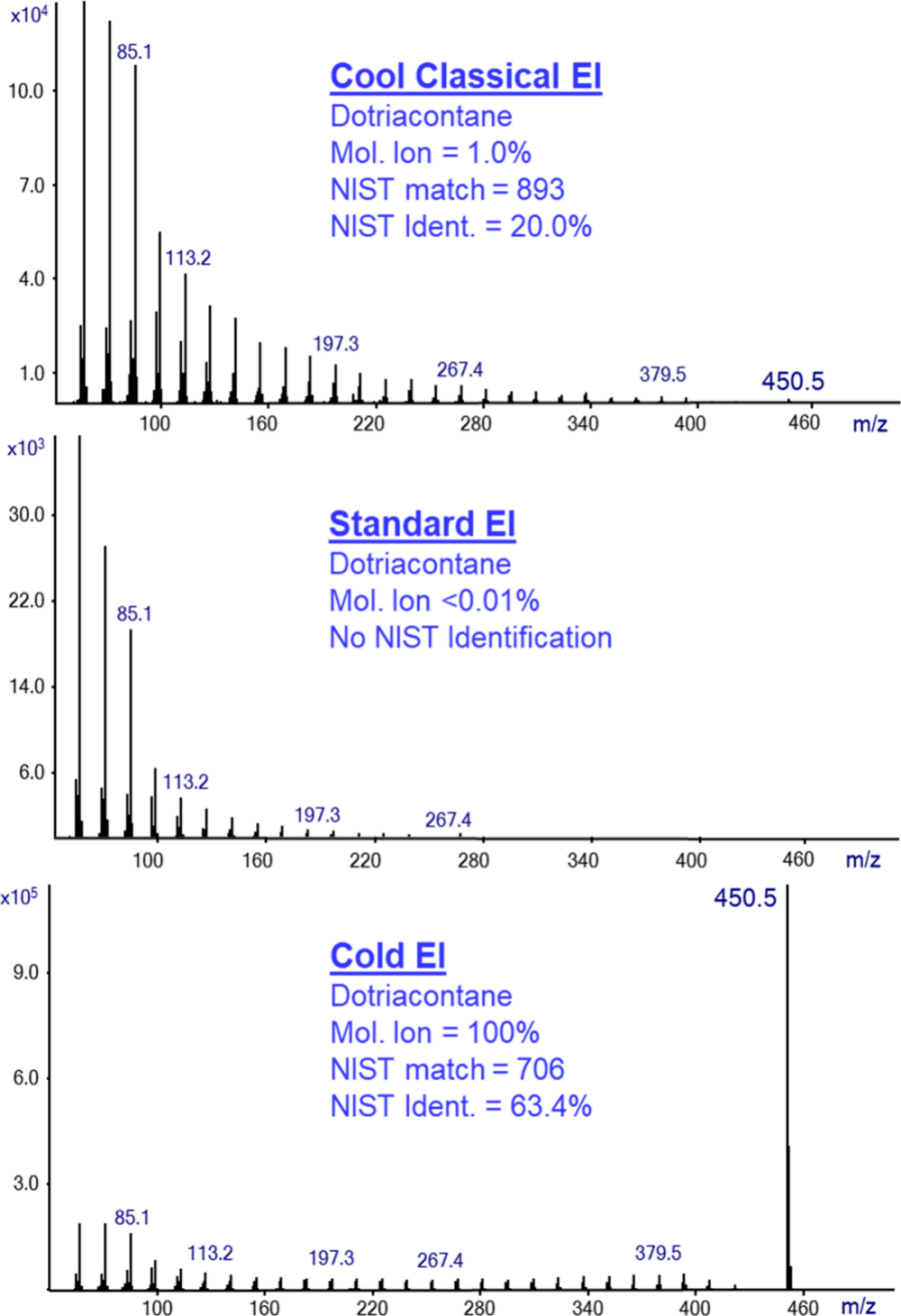
Mass spectra of dotriacontane (n-C_32_H_66_) in CCEI mode (upper trace), Standard EI (middle trace), and Cold EI (bottom trace). The figure shows that the NIST library in Standard EI fails to identify dotriacontane due to the lack of a molecular ion and the poor selectivity of the lower mass fragment ions. The CCEI mass spectrum shows a weak molecular ion with a small abundance of 1.0%. However, this is enough for the NIST library to identify the compound with good matches of 893 and 20.0% identification probability. Cold EI provides a dominant molecular ion with 100% abundance and a high identification probability of 63.4%.

Figure 5 shows the mass spectra obtained from the analysis of n-C_32_H_66_ (dotriacontane). As shown, the dotriacontane analysis using standard EI (middle trace) provided a very weak molecular ion with less than 0.01% relative abundance (barely detected in RSIM), and thus, the NIST library search fails to identify it. Furthermore, the standard EI mass spectrum was contaminated with column bleed ions and required background subtraction. The GC-MS analysis with Cold EI in the CCEI mode (upper trace) resulted in an enhanced molecular ion with 1.0% relative abundance. This is enough to achieve moderate identification with the NIST MS library, with a match factor of 893 and an identification probability of 20%. The same analysis using GC-MS with Cold EI in Cold EI mode delivers the best results with an enhanced molecular ion of 100% abundance and accordingly NIST identification probability of 63.4%. We consider this unique feature of Cold EI to be very important, since it paves the way for applications currently unavailable for GC-MS analysis or at all such as isomer distribution analysis.^16^ However, an important target of this paper is to show that Cool Classical EI provides similar appearance mass spectra to those of standard EI yet with better identification, which is a benefit of CCEI (d). We note that enhanced molecular ions further improve sample identification via the ability to convert the experimental isotope abundances into elemental formula with our TAMI software.^22^ This feature is of particular importance (essential) in the provision of elemental formulas for compounds that are not in the library.

Figure 5 also shows one of the most important benefits of GC-MS with Cold EI, both in CCEI mode and Cold EI mode, and that is its benefit (c) of an extended range of compounds amenable to GC-MS analysis, as hydrocarbons cannot be properly analyzed without exhibiting molecular ions.

**Figure 6:**
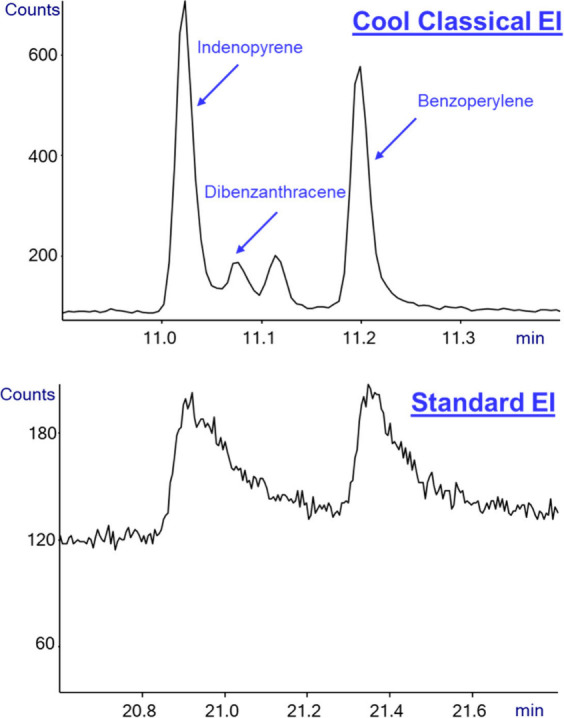
Mass spectra from the analysis of a PAH mixture in SIM mode at *m*/*z* = 276.1. The analysis using GC-MS with Cold EI in CCEI mode (upper trace) shows significantly better chromatographic separation and signal-to-noise ratio than that of GC-MS with standard EI (bottom trace). We also note that the analysis in CCEI is much more informative and provides additional data, such as revealing dibenzanthracene, which is not available when using GC-MS with standard EI.

As demonstrated and discussed in a few papers,^8,9,24^ Cold EI improves all the central performance aspects of GC-MS with standard EI and delivers new capabilities due to its unique SMB interface and contact-free fly through ion source. Since there is no interaction between the analytes and the metallic surface of the ion source, the analysis is much more informative. Figure 6 shows such new information from the analysis of 1 pg on-column each polycyclic aromatic hydrocarbons (PAHs) in a mixture (Retstek EPA 8270 mixture, Cat. Number 31995, Restek, Bellefonte, PA) in SIM mode of *m*/*z* = 276.1 for the analysis of indenopyrene, dibenzoanthracene, and benzoperylene. As shown in the bottom trace of Figure 6, the PAH chromatography exhibits severe intraion-source peak tailing and thus the exhibited signal-to-noise ratio in standard EI is very poor. Furthermore, this peak tailing leads into nonlinear signal dependence on the on-column amount, poor quantification RSD and high limit of detection (LOD) as further described and discussed in ref (25). However, the upper trace shows the same analysis using GC-MS with Cold EI in CCEI mode without any ion source related peak tailing and with a very good (much better than in standard EI) signal-to-noise ratio.

Moreover, the CCEI mass chromatogram contains additional valuable information about the exposure and finding of dibenzanthracene. We note that this information is unavailable when using GC-MS with Standard EI due to its peak tailing. Furthermore, the CCEI mass chromatogram exhibits an additional small peak after that of dibenzanthracene, which is from cholesterol carryover, as cholesterol has a major fragment ion at *m*/*z* = 275 with its isotopologue at *m*/*z* = 276. Thus, Cool Classical EI provides an additional benefit (e) of elimination of ion source related peak tailing as demonstrated in Figure 6.

**Figure 7:**
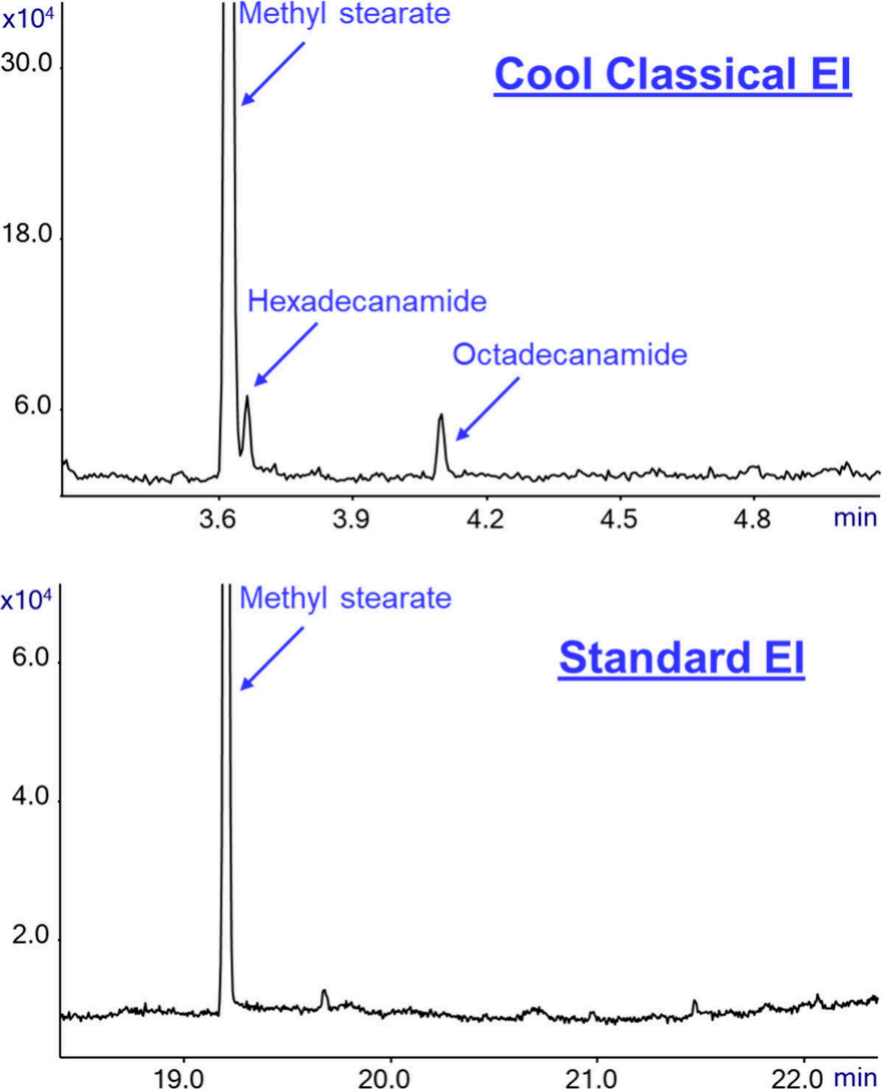
Impurity analysis in our standard test mixture of hexadecane, methyl stearate, cholesterol, and dotriacontane that was zoomed five times around the elution of methyl stearate. The figure shows that CCEI (upper trace) provides an additional two peaks and their identification information on hexadecanamide and octadecanamide, which are unavailable when using Standard EI.

Figure 7 shows the additional information that is provided by GC-MS with Cold EI in its CCEI mode while analyzing our test mixture. As shown, the mass chromatogram after the elution of methyl stearate shows peaks for both hexadecanamide and octadecanamide that were easily identified from the CCEI mass chromatogram by the NIT library with 81% and 70% identification probabilities. This information is unavailable when analyzing the same sample using GC-MS with Standard EI at the observed approximate level of 20–30 pg on-column amount, as these compounds are fully missing in standard EI without even exhibiting any single ion in the RSIM of the standard EI mass chromatogram. As known, compounds with OH or NH such as amides react particularly at low levels with the standard EI metallic ion source surface and thus degrade and require derivatization.^23,26,27^ Accordingly, Cool Classical EI also provides its benefit (f) of elimination of intra standard EI ion source degradation.

**Figure 8:**
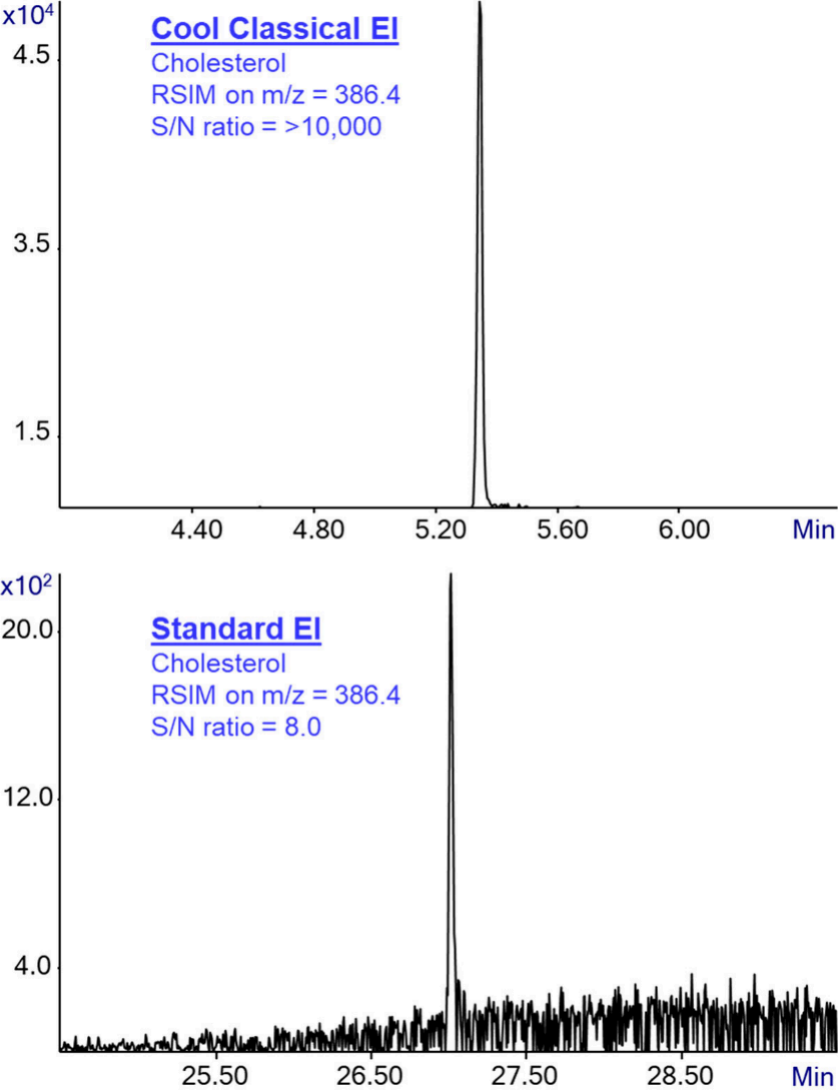
Extracted ion chromatogram (EIC) of cholesterol at its molecular ion *m*/*z* = 386.4, using CCEI (top trace) and standard EI (bottom trace). As shown in the figure, the signal-to-noise ratio of GC-MS with Cold EI in CCEI mode is >10000, which is dramatically better than the poor signal-to-noise ratio of 8 that was obtained using GC-MS with standard EI.

GC-MS with Cold EI in both CCEI and Cold EI modes also delivers much better signal-to-noise ratios than GC-MS with standard EI, as extensively described and discussed for Cold EI.^25^Figure 8 demonstrates the superior signal-to-noise ratio of CCEI in comparison with standard EI for cholesterol. The figure shows the extracted ion chromatogram (EIC) of the molecular ion of cholesterol at *m*/*z* = 386.1, using CCEI (upper trace) and standard EI (bottom trace). As shown, the signal-to-noise ratio for the standard EI cholesterol EIC is 8, while in the CCEI analysis there is zero baseline noise and thus it provides S/N > 10000. Like Cold EI, CCEI provides some vacuum background filtration due to having near zero intra ion source electric field, and thus, in view of the Agilent feature of software-based elimination of single ion noise unless it appears at neighbor dwell times we often obtain zero baseline noise as demonstrated in Figure 8.

Accordingly, we had zero CCEI baseline noise in all four test mixture compound mass chromatograms of hexadecane, methyl stearate, cholesterol, and dotriacontane. Furthermore, for dotriacontane, we had high signal and zero noise in CCEI (S/N > 10000) while we had near zero signal and high noise in standard EI (S/N = 1.1 in PTP). Thus, we found for all these compounds much better S/N in their EIC in CCEI than in standard EI. Accordingly, CCEI also provides its benefit (g) of the provision of better S/N than standard EI.

## Conclusions

In this paper, we show that GC-MS with Cold EI also includes a Cool Classical EI (CCEI) mode of operation that improves all of the major features of GC-MS with standard EI, yet it provided CCEI mass spectra that are similar to those obtained with standard EI ion sources. The CCEI mode provides the following main benefits in comparison with standard EI: (a) faster analysis, (b) uniform response, (c) extended range of compounds amenable for analysis, (d) improved sample identification, (e) elimination of standard EI ion source related peak tailing, (f) elimination of intra standard EI ion source degradation, and (g) provision of a much better signal-to-noise ratio. We note that CCEI shares with Cold EI other benefits that are not experimentally demonstrated in this article, such as superior linearity and much greater linear dynamic range. In ref (25) we demonstrated these Cold EI benefits for cholesterol and n-C_32_H_66_, and since they originate from the Cold EI use of a fly through ion source, it is easy to assume that they are shared by CCEI. These several unique benefits and features of Cool Classical EI pave the way for the analysis of compounds that otherwise cannot be analyzed using GC-MS.

Cool classical EI mode is obtained while operating the Cold EI interface and ion source at 10 mL/min combined column and makeup gas flow rate, and the transition from Cold EI to CCEI can be automated and even performed during the analysis.^9^

We demonstrated in this paper how GC-MS with Cold EI in its CCEI mode moderately enhances the molecular ion abundances and thus provides better identification via EI mass spectral libraries such as NIST, yet it retains high similarity (matching factors) to the standard EI mass spectra. We conclude that GC-MS with Cold EI in CCEI mode brings major benefits over standard GC-MS analysis while retaining the visual standard EI mass spectra that one is used to see, and thus, one would not challenge themselves to accept the correctness of the results. On the other hand, if one wishes to have the best results, GC-MS with Cold EI in its Cold EI mode is the best option for GC-MS analysis.
